# Leishmania donovani Exploits Tunneling Nanotubes for Dissemination and Propagation of B Cell Activation

**DOI:** 10.1128/spectrum.05096-22

**Published:** 2023-07-05

**Authors:** Tanja Stögerer, Sasha Silva-Barrios, Liseth Carmona-Pérez, Sharada Swaminathan, Linh Thuy Mai, Louis-Philippe Leroux, Maritza Jaramillo, Albert Descoteaux, Simona Stäger

**Affiliations:** a Institut National de la Recherche Scientifique (INRS) – Centre Armand-Frappier Santé Biotechnologie and Infectiopôle INRS, Laval, Quebec, Canada; George Washington University

**Keywords:** B cells, tunneling nanotubes, polyclonal B cell activation, *Leishmania donovani*, *Leishmania*

## Abstract

Polyclonal B cell activation and the resulting hypergammaglobulinemia are a detrimental consequence of visceral leishmaniasis (VL); however, the mechanisms underlying this excessive production of nonprotective antibodies are still poorly understood. Here, we show that a causative agent of VL, Leishmania donovani, induces CD21-dependent formation of tunneling nanotubule (TNT)-like protrusions in B cells. These intercellular connections are used by the parasite to disseminate among cells and propagate B cell activation, and close contact both among the cells and between B cells and parasites is required to achieve this activation. Direct contact between cells and parasites is also observed *in vivo*, as L. donovani can be detected in the splenic B cell area as early as 14 days postinfection. Interestingly, *Leishmania* parasites can also glide from macrophages to B cells via TNT-like protrusions. Taken together, our results suggest that, during *in vivo* infection, B cells may acquire L. donovani from macrophages via TNT-like protrusions, and these connections are subsequently exploited by the parasite to disseminate among B cells, thus propagating B cell activation and ultimately leading to polyclonal B cell activation.

**IMPORTANCE** Leishmania donovani is a causative agent of visceral leishmaniasis, a potentially lethal disease characterized by strong B cell activation and the subsequent excessive production of nonprotective antibodies, which are known to worsen the disease. How *Leishmania* activates B cells is still unknown, particularly because this parasite mostly resides inside macrophages and would not have access to B cells during infection. In this study, we describe for the first time how the protozoan parasite Leishmania donovani induces and exploits the formation of protrusions that connect B lymphocytes with each other or with macrophages and glides on these structures from one cell to another. In this way, B cells can acquire *Leishmania* from macrophages and become activated upon contact with the parasites. This activation will then lead to antibody production. These findings provide an explanation for how the parasite may propagate B cell activation during infection.

## INTRODUCTION

Leishmaniasis is a disease caused by obligate intracellular protozoan parasites of the *Leishmania* genus (1). These parasites predominantly infect macrophages, where the promastigote form differentiates into the amastigote form, which proliferates inside the mammalian host (2). The clinical manifestations of the disease are parasite species dependent and can range from self-curing infections characterized by lesions in the skin or mucosal membranes to severe tissue disruptions of visceral organs, which are fatal if left untreated. The life-threatening visceral leishmaniasis (VL) is caused by Leishmania donovani, among other *Leishmania* species, and results in the often-simultaneous enlargement of the liver and spleen in a condition termed hepatosplenomegaly, along with anemia, fever, and hypergammaglobulinemia (3).

Hypergammaglobulinemia has long since been identified as a characteristic symptom of VL. Characterized by abnormally elevated levels of immunoglobulins in the blood serum, it presents across all species susceptible to natural VL infection, including humans (4), dogs (5), and cats (6), as well as those used as experimental models, such as nonhuman primates (7), hamsters (8), and mice (9–11). This excessive production of nonprotective antibodies can closely resemble autoimmune conditions and exacerbates the disease through antibody-mediated pathology (12). Indeed, high antibody titers during L. donovani infection were previously proposed to be predictive of disease progression in humans (13). Nevertheless, our understanding of the mechanisms underlying this detrimental immune process is still limited.

Despite their central role in the production of antibodies, B cells have rarely been the focus of *Leishmania* research, and decades after the link between hypergammaglobulinemia and VL was drawn, the literature on the role of B cells in this disease remains sparse (14). Their contribution to the pathology of L. donovani infection was first identified in a study using a mouse model of visceral leishmaniasis, where B cells were shown to play a negative role and exacerbate the disease (15). Subsequent studies by our group and others identified polyclonal B cell activation and resulting hypergammaglobulinemia as the main route through which B cells exacerbate disease (9–11, 16). Indeed, *Aicda*^−/−^ mice, which are incapable of producing hypermutated and/or class-switched immunoglobulins, are highly resistant to L. donovani infection (11). Interestingly, both innate immune B cell activation through endosomal Toll-like receptors (TLRs) by *Leishmania* parasites and type I interferon (IFN-I) are required to promote hypergammaglobulinemia, as B cell-specific ablation of endosomal TLR signaling or the IFN-I receptor (IFNAR) resulted in severely decreased IgG titers upon L. donovani infection (10). Thus, IFNAR was shown to be involved in a positive feedback loop that resulted in the upregulation of endosomal TLRs and in enhancing the expression of various cytokines upon B cell exposure to *Leishmania* amastigotes (10).

In previous work from our group studying the early stages of B cell-parasite interaction, we found the parasite to induce the formation of membrane protrusions that branch out from the B cells (16). Rearrangements of the actin cytoskeleton leading to cell spreading and formation of short spike-like protrusions have been linked with B cell activation and, more specifically, B cell receptor (BCR) signaling (17), as well as antigen extraction and internalization (18); however, as the membrane protrusions observed between B cells exposed to L. donovani are both longer and more substantial than these described filopodia, their functional significance in the context of this infection remains to be discerned.

As *Leishmania* is an obligate intracellular parasite that mostly resides in macrophages, the question remains about how amastigotes come into direct contact with B cells during infection. We have previously reported that marginal zone B cells (MZB) were carrying the parasite a few hours after intravenous infection with amastigotes and suggested that MZB may shuttle from the splenic marginal zone into the B cell follicle and thus deliver the parasite to follicular B cells (16), but how MZB capture parasites is still unclear. Moreover, the importance of this direct interaction with the parasite for B cell activation and the dynamics underlying the communication between cells and the parasite remain to be fully elucidated.

In this study, we set out to investigate the dynamics of intercellular communication between B cells and Leishmania donovani amastigotes to better understand the pathways through which hypergammaglobulinemia is induced. We show that splenic naive B cells require direct contact with the parasite to become activated and that B cells can disseminate L. donovani amastigotes and thus activation among themselves with the help of CD21-induced formation of a tunneling nanotubule (TNT)-like membrane connection between cells. This interaction also results in an early and transient IFN-I wave induced in B cells by the parasite. Moreover, we show that L. donovani can be found in the splenic B cell area of infected mice as early as 14 days postinfection, just before the onset of hypergammaglobulinemia. We also show that parasites or parasite components can be transferred via TNT-like structures from macrophages to B cells, suggesting that MZB may receive amastigotes from infected macrophages and will then carry them to the B cell area.

## RESULTS

### B cells can transmit L. donovani amastigotes among each other to disseminate activation via direct contact with the parasite.

**FIG 1:**
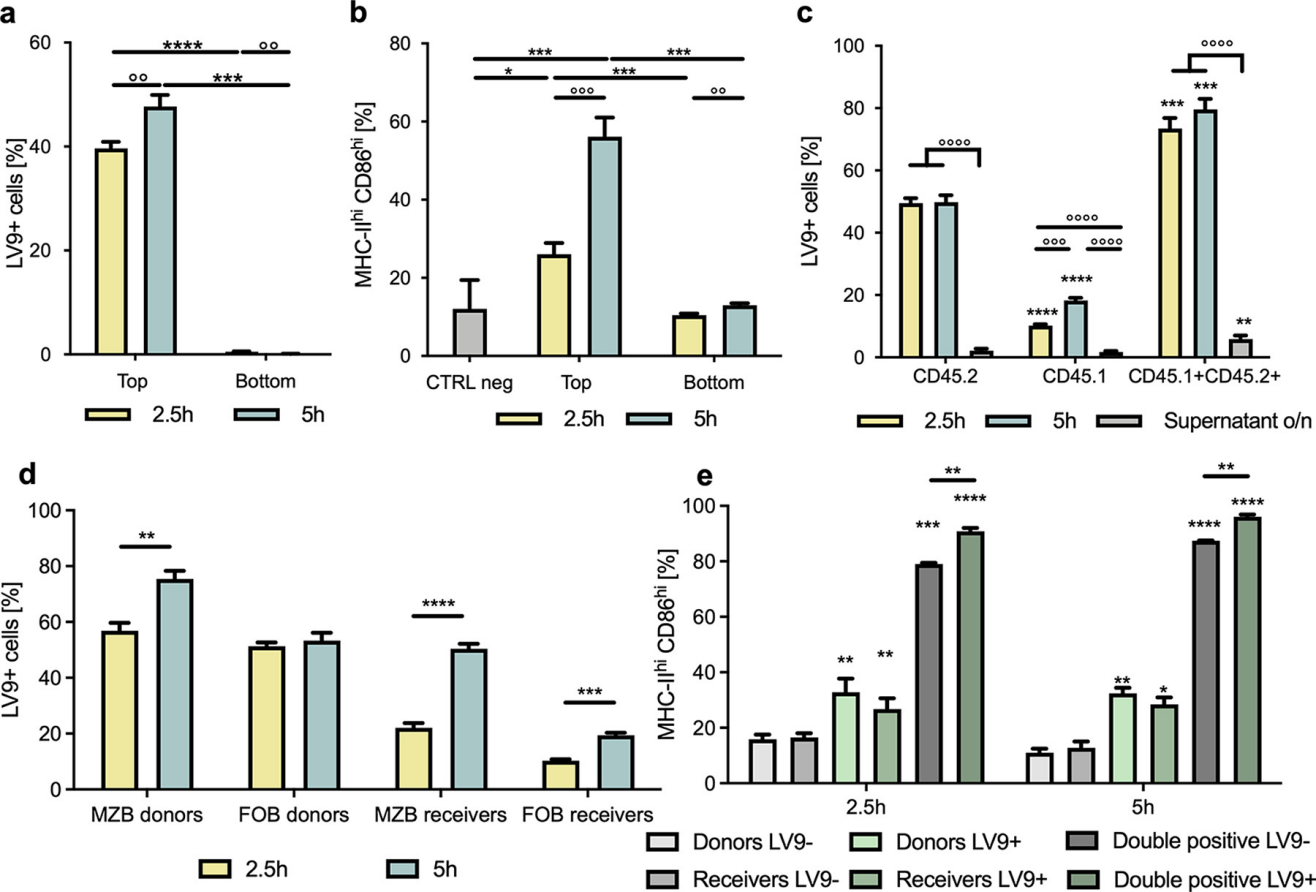
B cells can pass on L. donovani amastigotes among each other to disseminate activation via direct contact with the parasite. (a and b) Purified B cells exposed to *Rag1*^−/−^ mouse serum-coated L. donovani amastigotes (MOI, 1:10) were separated from naive B cells using permeable membrane inserts of insufficient pore size to allow for passage of parasite and exposed for 2.5 h or 5 h. (a) Percentage of cells carrying PKH67-stained amastigotes in the top and bottom compartments as assessed by fluorescence-activated cell sorting. (b) Percentage of cells expressing high levels of CD86 and MHCII in the top and bottom compartments compared to a nonexposed control. (c to e) B cells purified from CD45.2 mice were exposed to *Rag1*^−/−^ serum-coated L. donovani (MOI, 1:10) for 1 h before thorough washing to remove uncaptured parasite and exposure to naive B cells purified from CD45.1 mice for 2.5 h or 5 h. (c) Percentage of cells carrying PKH67-stained positive amastigotes in cells gated on their expression of CD45.1 or CD45.2 as measured by flow cytometry. (d) Percentage of MZB (CD21^hi^ CD23^lo^) and FoB (CD21^lo^ CD23^hi^) cells carrying parasite within the CD45.2^+^ donor and CD45.1^+^ receiver groups. (e) Percentage of cells expressing high levels of CD86 and MHCII within the CD45.2^+^ donors, CD45.1^+^ receivers and CD45.1^+^ CD45.2^+^ clusters carrying parasite (LV9^+^) or not (LV9-). Data represented as mean ± standard deviation from one of three independent experiments. *, *P* < 0.05; ** and °°, *P* < 0.01, * ** and °°°, *P* < 0.001, **** and °°°°, *P* < 0.0001.

We have previously reported that splenic B cells can capture L. donovani amastigotes upon *in vitro* exposure to the parasite (16) and that, following this interaction, the parasites sit in IgM-rich pockets on the B cell surface, leading to cell clustering and upregulation of the expression of major histocompatibility complex class II (MHCII) and activation markers such as CD86, followed by cell death within 24 to 48 h (10, 16). Thus, we first wanted to investigate whether direct contact between cells and between cells and parasites is required for B cell activation or if activation can simply be achieved through soluble mediators, such as cytokines. To this end, we separated primary splenic B cells using permeable membrane inserts with a pore diameter insufficient to allow for the passage of the amastigotes and found that indeed this hindered the transport of parasites from the upper, parasite-exposed compartment to the lower compartment to which no parasites were added initially (Fig. 1a). As L. donovani amastigotes isolated from mice are coated in complement C3 (16) and this coating is lost after thawing of frozen parasites, we incubated previously frozen amastigotes with serum from *Rag1*^−/−^ mice prior to their exposure to B cells to mimic their state *in vivo*. In the absence of the parasite in the lower compartment, these cells did not increase the expression of MHCII and activation markers such as CD86 at either 2.5 or 5 h postexposure, while cells in the top compartment, able to establish contact with the parasite, showed a significant increase of the proportion of MHCII^hi^ CD86^hi^ B cells at both time points (Fig. 1b). The lack of upregulation of these markers in the cells physically separated from the parasite and from cells carrying amastigotes indicates that contact with the parasite or with cells carrying *Leishmania* is indeed required to activate B cells and that soluble mediators alone are insufficient to propagate activation.

Next, we wanted to see if the parasite could be passed on from one cell to another as a possible explanation for how activation is disseminated among B cells. To this end, we separately exposed B cells from C57BL/6 mice (CD45.2) to *Rag1*^−/−^ mouse serum-coated L. donovani amastigotes for 1 h before thoroughly washing the cells to remove any uncaptured parasite and subsequently incubating them with naive B cells purified from CD45.1 congenic mice. Indeed, about 10% of CD45.1 B cells (receivers) were carrying parasites at 2.5 h (Fig. 1c) and 20% were at 5 h of coculture (Fig. 1c).

To exclude the possibility that the parasite can simply detach from the B cells and naive B cells can then recapture amastigotes from the supernatant, we exposed cocultured naive CD45.1 and CD45.2 B cells to the supernatants collected from exposed cells and found negligible capture of the parasite by these fresh cells. This indicates that B cells can indeed pass on parasites from one cell to another by means other than a simple detachment-reattachment mechanism. Strikingly, we also observed a small population of CD45.1^+^ CD45.2^+^ double-positive cells, pointing toward clustering between initially exposed (donors) and nonexposed (receivers) cells, almost all of which stained positive for the parasite. Within both the donor and receiver groups, we found a higher percentage of marginal zone B cells (MZB) than of follicular B cells (FoB) to carry the parasite, although MZB make up only around 5% of splenic B cells, with FoB representing the majority of B cells with about 90 to 95% (see Fig. S1a in the supplemental material). This points toward a stronger involvement of MZB in the capture or transfer of parasites (Fig. 1d), which is in agreement with our previous observations (16).

When looking at the activation status of these cells, we found that the presence of parasites on both initially exposed and receiving cells led to an upregulation of the expression of MHCII and CD86 (Fig. 1e). Interestingly, the upregulation of the expression of these markers in the clustering cells was even more pronounced, indicating the existence of two possible levels of activation induced by the capture of parasite and clustering of the cells. Additionally, clustered cells kept their high expression of CD86 and MHCII even when no parasite was detected in the clusters, which could point toward the cells keeping their activation status even after passing on the parasite. Expression of the different CD45 isoforms did not affect the cells’ capacity to capture or pass on the parasite, as these observations are mirrored in experiments initially exposing CD45.1 cells and subsequently coculturing them with CD45.2 B cells (Fig. S1b to d). Taken together, our results suggest that B cells are capable of transferring L. donovani amastigotes among each other and that direct contact between B cells and the parasite as well as B cell clustering is required for activation.

### Amastigotes disseminate among B cells via tunneling nanotubule-like protrusions.

**FIG 2:**
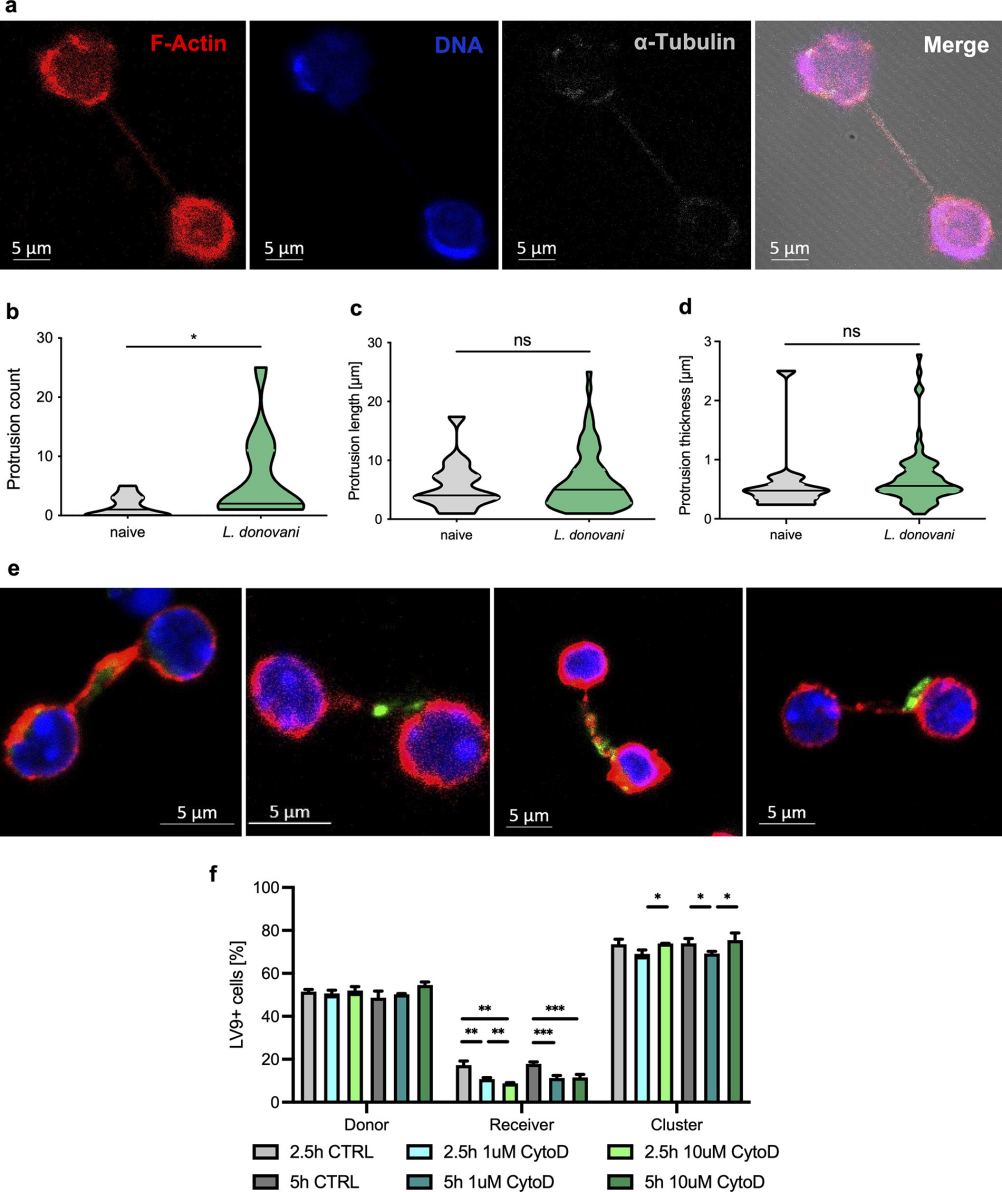
L. donovani-exposed B cells increase the formation of tunneling nanotubule-like protrusions that participate in the intercellular transfer of the parasite. B cells were incubated for 2 to 5 h with or without the presence of fluorescently labeled L. donovani (MOI, 1:10). (a) Naive B cells were labeled using phalloidin-AF594 (F-actin, red), Hoechst 33342 (blue), and anti-tubulin-AF647 (gray). (b to d) Protrusions between B cells with or without parasite were counted over 70 microscope fields per sample (b) and measured for their length (c) and thickness (d) using Zen 2011 software (Zeiss). (e) Representative images of TNT-like connections made of actin (phalloidin-AF594, red) carrying PKH67-labeled L. donovani amastigotes (green). Nuclei were stained using Hoechst 33342 (blue). (f) B cells purified from CD45.1 mice were exposed to L. donovani (MOI, 1:10) for 1 h before thorough washing to remove uncaptured parasites and treatment with 1 or 10 μM cytochalasin D, prior to coincubation with CD45.2^+^ B cells for 2.5 or 5 h. Percentage of cells carrying PKH67-stained amastigotes in cells initially exposed to parasite (donor), nonexposed cells (receiver), and clusters between the two types as measured by flow cytometry. Data represented as mean ± SD from three independent experiments. *, *P* < 0.05; **, *P* < 0.01; ***, *P* < 0.001; ****, *P* < 0.0001; ns, not significant.

Subsequently, we sought to investigate the mechanism by which B cells pass on L. donovani amastigotes to each other. As previously reported (16), we found B cells exposed to the parasite to form long membrane protrusions (Video S1). Interestingly, these protrusions can be seen to establish connections between two cells. To gain a better understanding about their functional role and significance, we thus set out to characterize these structures. Interestingly, we found them to be primarily F-actin based with smaller amounts of tubulin (Fig. 2a), which is in line with literature descriptions of tunneling nanotubule (TNT)-like protrusions (19, 20). Remarkably, we found the incidence, but not the length or thickness, of these connections to be significantly increased among cells exposed to L. donovani amastigotes (Fig. 2b to d). Of note is that the seemingly low incidence of connecting protrusions is owed to the mode of quantification of links formed between cells in a two-dimensional plane at a fixed point in time. This likely leads to an underestimation of the number of connections, as their formation can be seen as a much more frequent and dynamic, albeit difficult to reliably quantify, process in live-cell microscopy videos.

Intrigued by the induction of the formation of these B cell protrusions by the parasite, we set out to investigate whether these amastigote-induced protrusions could serve any functional purpose. Previous studies have demonstrated TNTs and TNT-like protrusions formed by other cell types to be exploited for disease propagation by bacteria (21–23) and viruses (24–26), so we wanted to know whether these protrusions could be used by L. donovani to pass between cells and hence propagate activation. Indeed, we frequently observed the presence of L. donovani amastigotes on the protrusions formed between two B cells (Fig. 2e), indicating that they could be used to allow gliding of the parasite from one cell to another, thus aiding in the dissemination of L. donovani between cells, which could result in polyclonal B cell activation and ultimately hypergammaglobulinemia (Fig. 2e).

Having thus identified induction of TNT-like protrusions by L. donovani amastigotes, we then wanted to investigate whether these intercellular connections could participate in the exchange of parasites among B cells. To this end, we similarly exposed B cells from CD45.1 mice to serum-coated amastigotes for 1 h and then thoroughly washed them until no free parasite was observed in the medium. Prior to coculture with initially nonexposed CD45.2^+^ B cells for 2.5 and 5 h, however, we incubated the cells with the potent actin polymerization inhibitor cytochalasin D at 1 or 10 μM, which has been previously described to inhibit formation of nanotubules (27, 28). This treatment did not negatively impact cellular viability after 5 h of incubation (Fig. S2a), although exposure of cells to the parasite decreased the viability of the B cells as previously published (16). Likewise, treatment with cytochalasin D did not interfere with capture of parasites by donor B cells or with cluster formation (Fig. 2f); however, the presence of parasite on initially nonexposed (receiver) cells significantly decreased after treatment with both 1 μM and 10 μM cytochalasin D (Fig. 2f), indicating a considerable involvement of TNT-like protrusions in the transfer of L. donovani amastigotes between B cells.

We next set out to investigate whether the formation of these protrusions was strictly amastigote specific or could also be induced by different parasite forms and species. Indeed, exposure of B cells to both axenically cultured L. donovani promastigotes (Video S2) and Toxoplasma gondii tachyzoites (Video S3) failed to promote the formation of similar membrane connections; however, opsonizing the promastigotes with serum from *Rag1*^−/−^ mice before coincubation with B cells conferred on the parasites the ability to induce clustering (Video S4) and the formation of TNT-like structures among the B cells (Fig. S2b and Video S5). This indicates that the serum coating on L. donovani amastigotes plays an important role in the induction of TNT-like protrusions in B cells.

### Cross-linking of complement receptor 2 induces protrusion formation.

**FIG 3:**
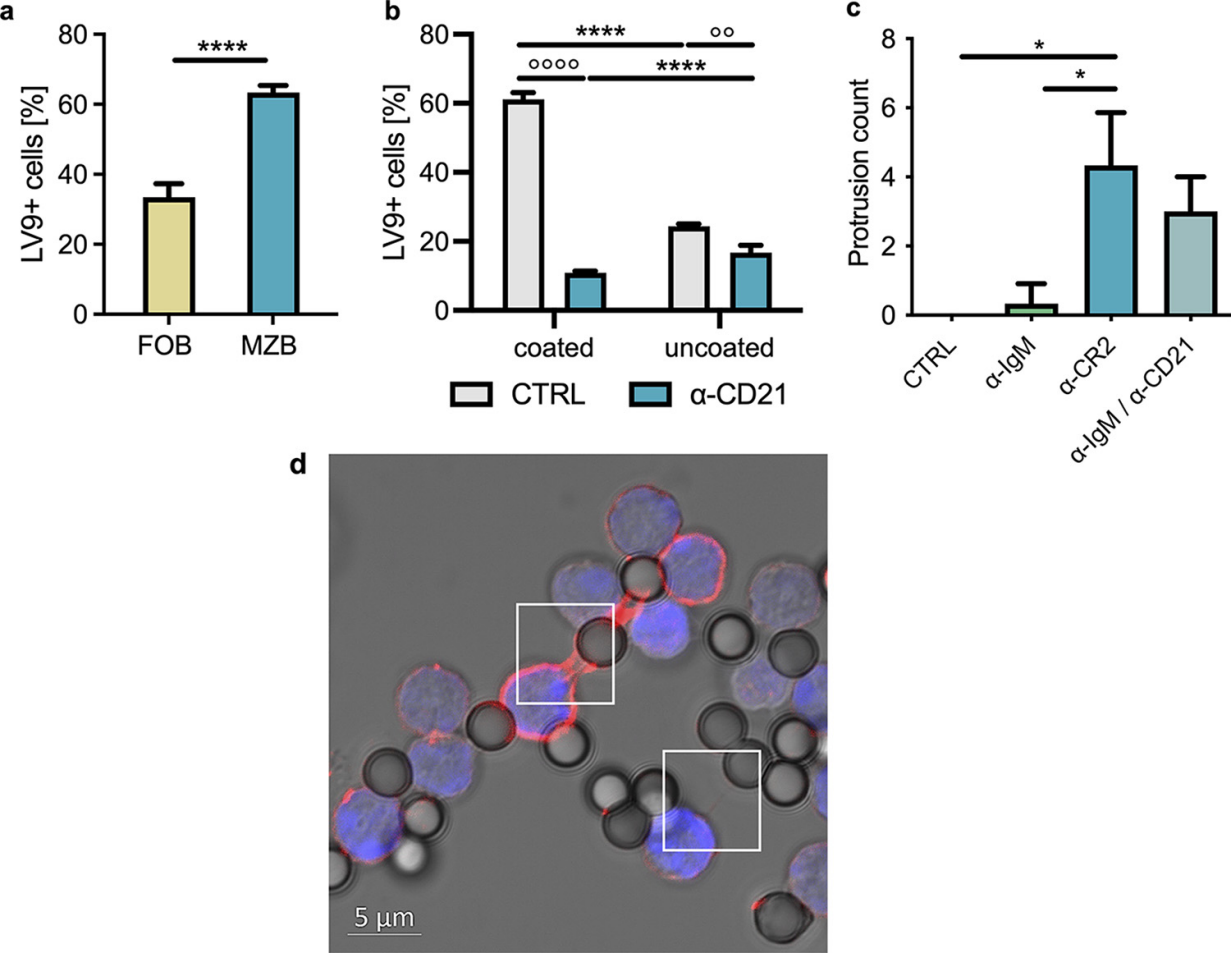
Complement receptor 2 mediates the capture of L. donovani by B cells and induces protrusion formation. (a) Purified B cells were exposed to L. donovani (MOI, 1:10) for 2.5 h, and the percentage of cells carrying PKH67-labeled parasite was assessed within MZB (CD21^hi^ CD23^lo^) and FoB (CD21^lo^ CD23^hi^) using flow cytometry. (b) B cells were incubated with CD21 blocking antibody 2 h prior to exposure to parasite that was previously frozen in liquid nitrogen (LV9 uncoated) or treated with fresh *Rag1*^−/−^ serum after thawing (LV9 coated). Percentage of cells carrying parasite was measured using flow cytometry. (c and d) Antibodies against IgM, CD21, or a combination of the two were coupled to latex beads and exposed to B cells for 5 h. (c) Protrusions between B cells with or without parasites were counted over 70 microscope fields per sample. (d) Representative image of B cells stained using phalloidin-AF594 (F-actin, red) and Hoechst 33342 (blue) forming protrusions with beads. Data represented as mean ± standard deviation from three independent experiments. *, *P* < 0.05; °°, *P* < 0.01, **** and °°°°, *P* < 0.0001.

MZB are the primary B cell subset seen to capture parasites *in vivo* (16) and are also more prone to interact with L. donovani amastigotes than follicular B cells (FoB). Indeed, about 65% of MZB compared to 35 to 40% FoB were seen to carry L. donovani amastigotes 2.5 h after exposure to the parasite (Fig. 3a), despite the fact that only about 5% of the B cells are MZB.

A key difference between MZB and FoB is their expression of complement receptor 2, also known as CD21, which is highly expressed in MZB and to a much lower degree in FoB (Fig. S3a) and serves as a distinguishing marker between the two subsets. Interestingly, we have previously found the L. donovani amastigotes isolated from *Rag1*^−/−^ mice to be coated in complement C3 (16), whose fragments are known to be ligands for CR2 (29). Therefore, we set out to examine whether CD21 plays a role in capturing L. donovani amastigotes by the B cells. To this end, we incubated thawed amastigotes with serum from *Rag1*^−/−^ mice to mimic the C3 coating of parasites *in vivo* (Fig. S3b) and exposed them to cells incubated with a CD21 (CR2) blocking antibody. Indeed, a blockade of surface CR2 on B cells dramatically decreased the capacity of the cells to capture serum-coated parasites, while the blockade affected attachment of uncoated parasites to a much lower degree (Fig. 3b), solidifying the role of CD21/CR2 in the interactions at the interface between B cells and L. donovani amastigotes.

We then set out to elucidate which surface receptor interactions might be responsible for the induction of observed protrusions formed by the B cells. As the B cell receptor (BCR) is known as a central receptor for B cell activation, we investigated cross-linking the BCR using an anti-IgM antibody in addition to the abovementioned CR2 for its ability to induce protrusions in B cells. To this end, we coupled antibodies for either the BCR (anti-IgM), CD21, or a combination of the two to latex beads and incubated them with naive B cells to examine them for any induction of TNT-like protrusions by surface receptor cross-linking. Remarkably, cross-linking CD21 on B cells gave rise to a significant increase in protrusion formation compared to cells exposed to uncoated beads, while cross-linking the BCR using anti-IgM-coupled beads did not lead to a significant induction of TNT-like protrusions (Fig. 3c and d). Similarly, coincubating B cells with beads coupled to a rat IgG2a isotype control antibody did not give rise to TNT-like protrusions (Fig. S3c). Taken together, this points toward a role of CD21 (CR2) rather than the BCR in the formation of these structures. Looking at L. donovani-induced protrusion formation in the two main splenic B cell subpopulations, we further identified the CD21^hi^ MZB as the primary subset responsible for TNT-like protrusions (Video S6), as opposed to the CD21^lo^ FoB (Video S7). Taken together, our results evidence an important role of CR2 in the induction of membrane protrusions by L. donovani amastigotes in B cells and in capturing parasites by B cells.

### Leishmania donovani induces the production of an early IFN-I in B cells.

**FIG 4:**
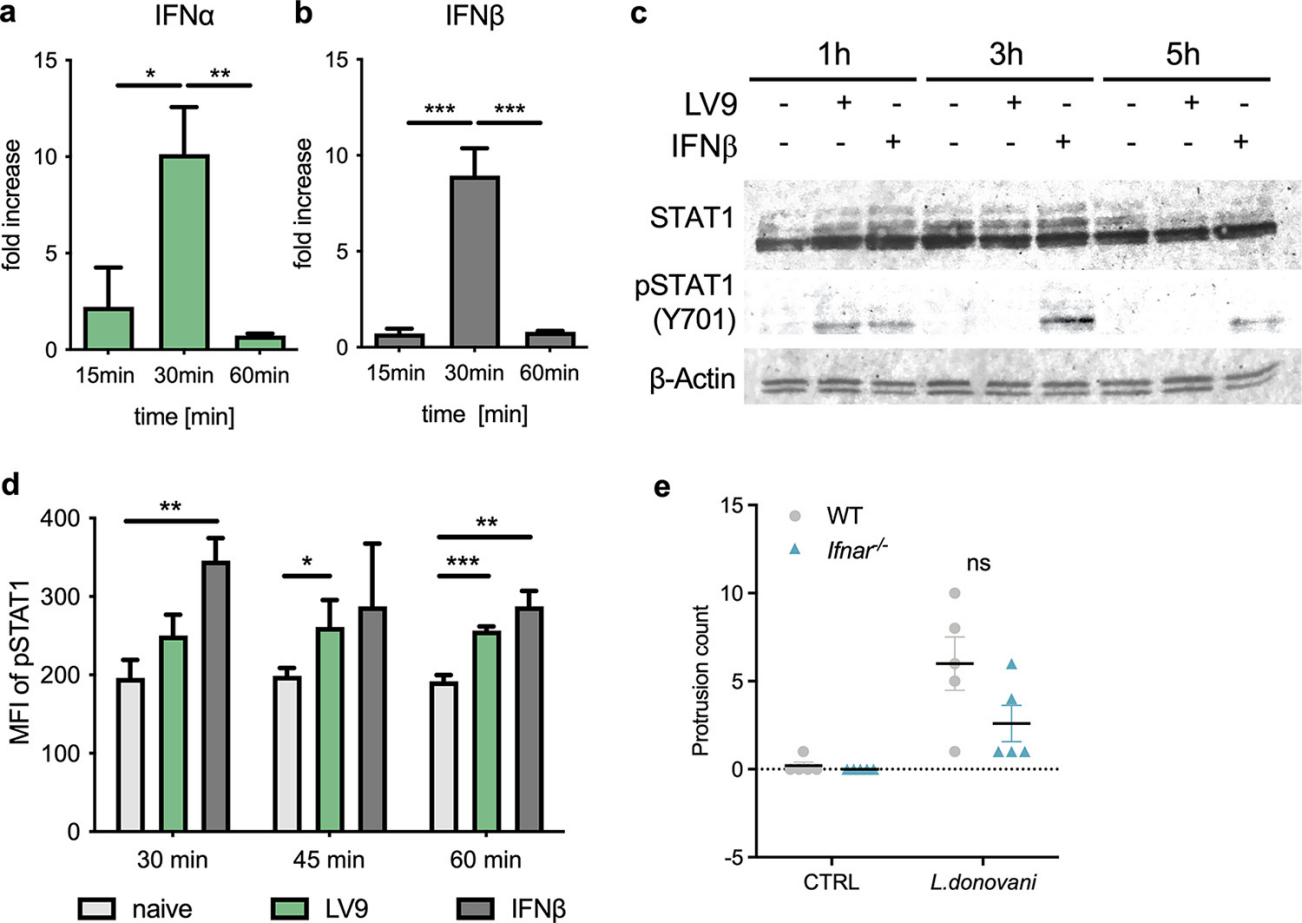
L. donovani induces a transient production of IFN-I leading to autocrine signaling in B cells. Purified B cells were exposed to L. donovani amastigotes (MOI, 1:10) or recombinant IFN-β as a positive control. (a and b) Fold change of IFN-α (a) and IFN-β (b) mRNA expression in splenic B cells exposed to L. donovani relative to unexposed B cells as measured by RT-qPCR. (c) Protein expression of STAT1 and p-STAT1 (pY701) using β-actin as a loading control. (d) Mean fluorescence intensity (MFI) of p-STAT1 expression (pY701) as assessed by flow cytometry. (e) B cells from control (C57BL/6) or *Ifnar1^−/−^* mice were incubated for 2 to 5 h with or without the presence of fluorescently labeled L. donovani. Protrusions between B cells with or without parasite were counted over 70 microscope fields per sample. Data represented as mean ± standard deviation from one of three to four independent experiments. WT, wild type; ns, not significant; *P* < 0.05; **, *P* < 0.01; ***, *P* < 0.001.

In addition to the induction of TNT-like protrusions by cross-linking of CR2, other pathways may also promote the formation of these connections. In previous studies, IFN-α has been identified to increase TNT formation in the human chronic myeloid leukemia cell line Kcl-22 (30), and our group has previously published an induction of IFN-I in B cells 8 h after exposure to the parasite (10). Thus, we sought to measure whether the parasite could also induce type I IFN at time points preceding the period in which we observe most connections to be formed, between 1 and 5 h after exposure. Indeed, we found that exposure of B cells to L. donovani amastigotes induces a transient expression of *Ifna* and *Ifnb* mRNA at 30 min after exposure of the B cells (Fig. 4a and b). This upregulation of *Ifna* and *Ifnb* mRNA was curtailed as it was no longer detectable at 60 min after exposure to the parasite. Concurrently, we observed that this induced production of IFN-I leads to intracellular signaling through phosphorylation of STAT1, a key player of the JAK/STAT pathway, at 1 h using Western blotting (Fig. 4c), as well as significant Y701 phosphorylation at both 45 min and 60 min in flow cytometry (Fig. 4d). Interestingly, although not statistically significant, the lack of type I IFN signaling in *Ifnar1*^−/−^ mice led to a lower formation of protrusions (Fig. 4e), which could be only partially explained by a minimal, yet significant, reduction in CD21 expression levels observed in both *Ifnar^−/−^* FoB and MZB compared with wild-type cells (Fig. S4a and b). This suggests that, in addition to inducing the upregulation of endosomal TLRs and amplifying hypergammaglobulinemia (10), IFN-1 may also be involved in the formation of TNT-like protrusions and the subsequent early parasite dissemination among B cells.

### L. donovani amastigotes are present in the splenic B cell area of infected mice.

**FIG 5:**
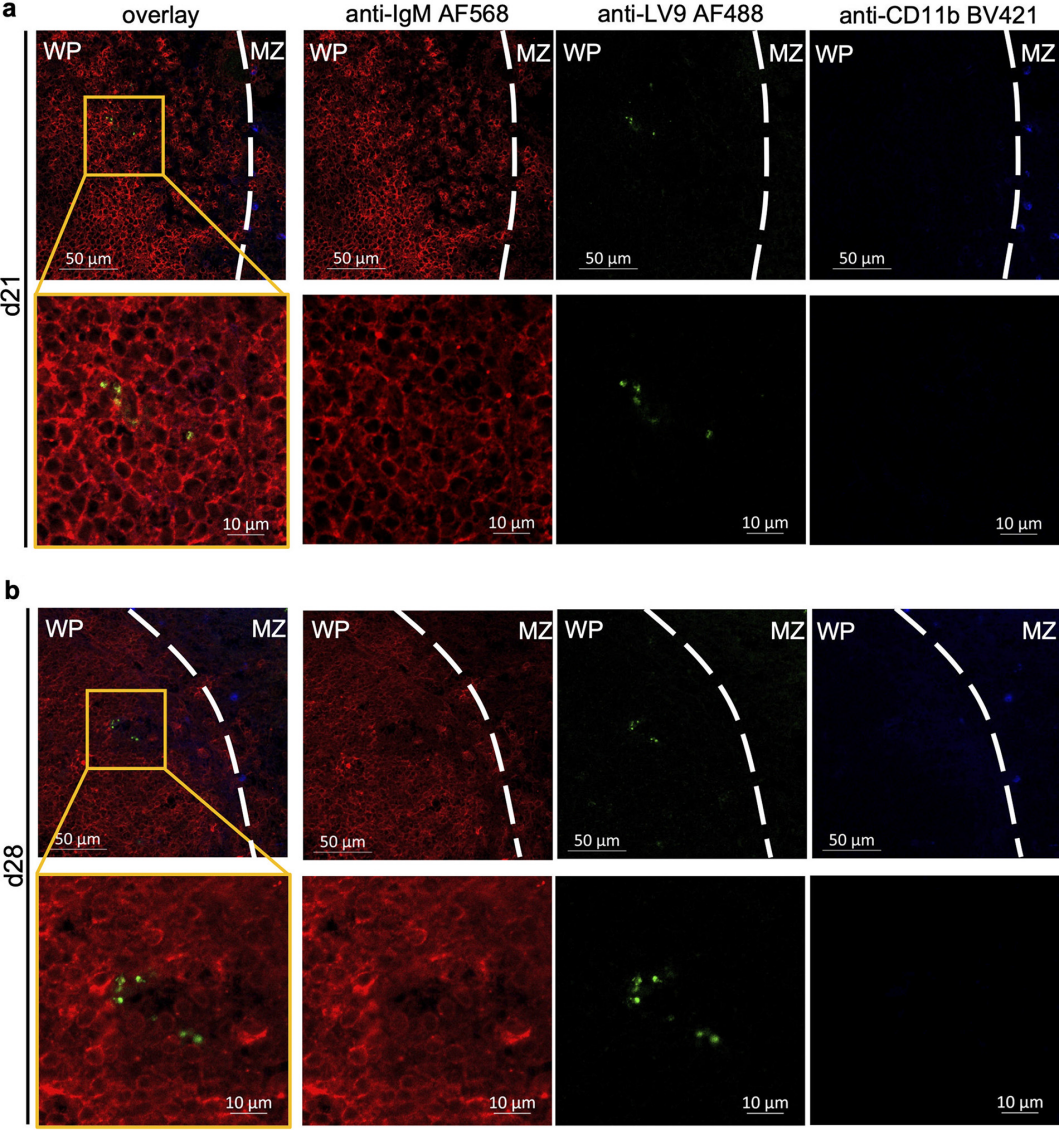
L. donovani is present in the splenic B cell area of infected mice. Splenic cryosections of L. donovani-infected mice at days 21 (a) and 28 (b) postinfection. The first row of each panel was taken using a 40× oil immersion objective while the second row represents an inset taken using a 63× objective at the location specified using a yellow square. Tissue sections were stained using IgM-AF568 (red) and BV421-conjugated CD11b (blue), and the parasite was stained by incubation with serum from L. donovani-infected hamsters and secondary anti-hamster-AF488 antibody (green). Abbreviations: MZ, marginal zone; WP, white pulp.

Having now characterized the interactions of B cells and L. donovani *in vitro*, we then set out to investigate whether such interactions also take place *in vivo*. In fact, despite macrophages being regarded as the primary target of L. donovani infection (2), previous work from our laboratory demonstrated that B cells isolated from infected mice can be seen to carry the parasite *ex vivo* (16). As this interaction could occur following release of parasite during tissue homogenization, we generated cryosections of the spleens of mice infected with L. donovani at various time points postinfection, and we found L. donovani to be present in the B cell area as early as day 14 (Fig. S5), with the number of parasites increasing toward later stages of disease (Fig. 5a and b). These results definitively show that B cells are in direct contact with L. donovani *in vivo* and thus could be activated through this direct interaction with the parasite.

### L. donovani amastigotes can be transferred between macrophages and B cells via TNT-like protrusions.

**FIG 6:**
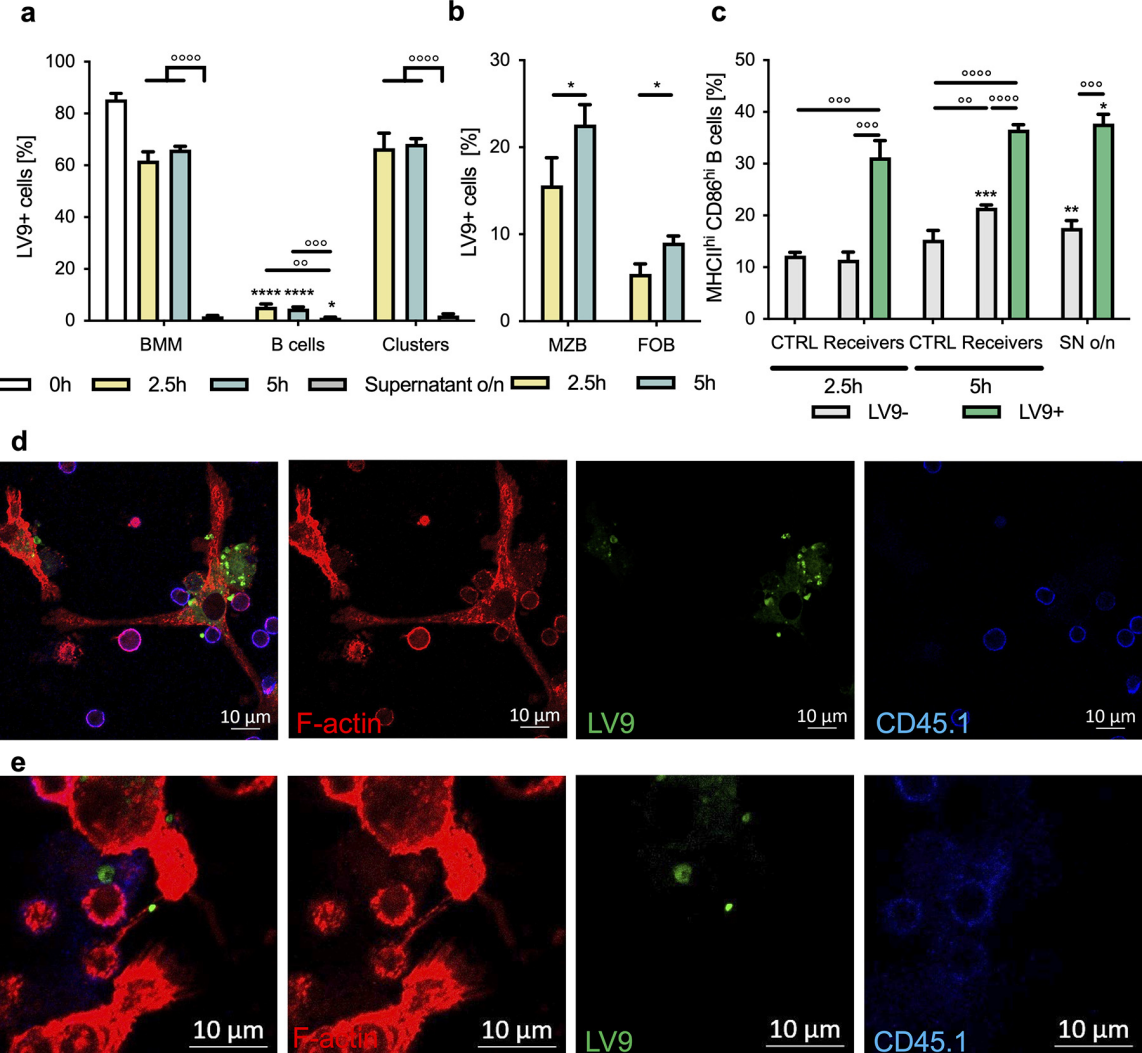
L. donovani amastigotes can be transferred between macrophages and B cells via TNT-like protrusions. Macrophages derived from the bone marrow of CD45.2 mice (BMM) were exposed to L. donovani (MOI, 1:10) overnight before thorough washing to remove uncaptured parasite and exposure to naive B cells purified from CD45.1 mice for 2.5 h or 5 h, at a 1:1 ratio. (a) Percentage of cells carrying PKH67-stained amastigotes in cells separated into CD45.1^+^ B cells, CD45.2^+^ BMM, or CD45.1^+^ CD45.2^+^ clusters as measured by flow cytometry. (b) Percentage of MZB (CD21^hi^ CD23^lo^) and FoB (CD21^lo^ CD23^hi^) carrying parasite within the initially nonexposed CD45.1^+^ B cells. (c) Percentage of B cells expressing high levels of CD86 and MHCII separated into cells carrying parasite (LV9^+^) and cells not in contact with the parasite (LV9-). SN o/n, supernatant from overnight cultures. (d and e) Representative confocal microscopy images of BMM infected with PKH67-labeled L. donovani amastigotes (green) and cocultured with naive B cells for 5 h. Immunofluorescence staining using phalloidin-AF594 (F-actin, red) and CD45.1 (B cells, blue). Data represented as mean ± standard deviation from one of four independent experiments. *, *P* < 0.05; ** and °°, *P* < 0.01, *** and °°°, *P* < 0.01, **** and °°°°, *P* < 0.0001.

As we have now verified that L. donovani can indeed be found in contact with B cells *in vivo*, we sought to investigate how the parasite might come to enter the B cell area in the first place. As macrophages represent the primary infection target for *Leishmania* (2) and are in close proximity to B cells in the marginal sinus of the spleen (31), we then chose to look at whether these different types of immune cells are capable of transferring L. donovani amastigotes among each other. Strikingly, we observed that even in the presence of macrophages, B cells still capture a significant number of amastigotes, albeit to a much lower degree than macrophages, when incubated together *in vitro* (Fig. S6a). To see whether L. donovani-infected bone marrow-derived macrophages (BMM) can indeed transfer parasites to B cells, we differentiated macrophages from congenic CD45.2 mice and infected them with L. donovani amastigotes overnight, and after thorough washing to remove uncaptured parasite, we cocultured these BMM with fresh B cells purified from CD45.1 mice. After both 2.5 and 5 h of coculture, a significant percentage of B cells stained positive for the parasite, indicating the transfer of L. donovani amastigotes from macrophages to B cells (Fig. 6a). The concomitant decrease of the percentage of infected macrophages after time point 0 (prior to the coincubation with B cells) is likely due to both loss of parasites to B cells and clustering (Fig. 6a). Within the B cells staining positive for L. donovani, MZB had a higher affinity for capturing the parasite at both time points than FoB (Fig. 6b). Additionally, low levels of clusters between B cells and macrophages can be observed, which could point toward possible surface interactions between the two cell types. These heterogenous clusters carry parasites to a high degree and can also be observed during confocal microscopy of a coculture of both immune cell types (Fig. 6d and e). Similar to transfer experiments between B cells, exposing fresh macrophages and B cells to the supernatants collected from exposed cells led to negligible capture of parasite by these fresh cells, indicating that this transfer of cells is not due to capture of free parasite from the medium (Fig. 6a). As expected, B cells that received L. donovani amastigotes from macrophages also upregulated MHCII and CD86, indicating activation (Fig. 6c). Interestingly, these markers were also slightly but significantly upregulated in B cells exposed to the L. donovani-carrying BMM which were not carrying parasite at the time of measurement, which may be due to loss of parasite after activation. One possible mechanism of loss of parasite besides the transfer of amastigotes to other naive B cells could be the reverse transfer of parasite from the B cells to BMM. Indeed, we found that parasites could also be transferred from B cells to macrophages, as initially nonexposed macrophages can be seen to carry substantial amounts of parasite after both 2.5 and 5 h of coculture with B cells initially exposed to L. donovani amastigotes (Fig. S6b). To determine whether whole parasites or parasite components were transferred between macrophages and B cells, we performed similar experiments, where we directly seeded and infected BMM on confocal microscopy slides and, after thorough washing, cocultured them with fresh B cells. As with flow cytometry, we saw capture of parasite by B cells, indicating that transfer indeed occurred (Fig. 6d). On these slides, all parasites were observed to be bound by either B cells or macrophages, further solidifying that the measured transfer is not due to capture of free parasite from the medium. Strikingly, we also observed the formation of L. donovani amastigote-carrying TNT-like protrusions between B cells and macrophages (Fig. 6e), which lays grounds for a possible role of this route of intercellular communication in this transfer.

## DISCUSSION

To date, the mechanisms underlying the detrimental polyclonal B cell activation observed during visceral leishmaniasis caused by Leishmania donovani are still poorly understood. This study identifies the formation of tunneling nanotubules between B cells and the consequent cluster formation as a possible mechanism through which these cells disseminate L. donovani among each other, spread activation, and contribute to polyclonal B cell activation. Moreover, we propose that MZB acquire parasites or parasite components from infected macrophages via TNT-like protrusions and then shuttle to the splenic B cell area, where FoB will then become activated by the parasite.

While we have previously shown that B cells purified from the spleens of mice infected with L. donovani can be seen to be in direct contact *ex vivo* (16), the significance of this direct contact between cell and parasite for B cell activation has not been previously studied, as there could be soluble messengers, such as extracellular vesicles and cytokines, present, which might mediate the spread of activation among B cells. In fact, *Leishmania* parasites are well known to be able to spontaneously shed extracellular vesicles or exosomes, both in their amastigote, in their promastigote, and in their metacyclic form (32–34). These vesicles are typically 50 to 150 nm in size and have been assigned various immunomodulatory functions in leishmaniasis, including the modulation of cytokine production and the regulation of intracellular signaling pathways (33, 35). While not much about the action of *Leishmania*-derived extracellular vesicles on B cells is known to date, there is previously published evidence that exosomes spontaneously shed from Leishmania amazonensis promastigotes are able to activate peritoneal B-1 cells to produce interleukin-6 (IL-6) and tumor necrosis factor (TNF) (36). Another group of soluble mediators known both to be produced by and to act on B cells is cytokines. Indeed, the production of cytokines such as IL-10, IL-6, and IFN-I by different splenic B cell subsets in response to L. donovani has been demonstrated in multiple studies (9, 10, 37). Despite choosing permeable membrane inserts of a pore size sufficient to allow for the passage of cytokines, as well as vesicles substantially bigger than the average size of shed particles reported in the literature, however, we see no increase in activation in the naive B cells that only are exposed to any soluble mediators but cannot establish direct contact with the parasite, indicating that in the case of B cell activation by L. donovani amastigotes, direct interaction of the parasite with naive B cells is required for their activation.

In addition to direct contact with the parasite, we find expression of MHCII and activation markers such as CD86 to be even further increased in B cells forming clusters among each other. This could possibly be explained through interactions at the surface of B cells. Indeed, B cells have been proposed to be able to expand the number of antigen-binding B cells by transfer of BCR via close-contact membrane exchange with bystander B cells, which is increased in cells undergoing BCR activation (38). Hence, the increased activation in B cell clusters could be due to the transfer of BCR between B cells. Another possible mechanism of how these clusters could elevate activation above the level observed in singlet B cells in contact with the parasite could be through surface receptor interactions, as upregulation of CD86 and MHCII by B cells has been linked to increased antigen presentation (32); however, antigen presentation by B cells has been found not to greatly contribute to the exacerbation of VL (9), making it unlikely that this represents a major mechanism of B cell activation in the course of this disease.

In this study, we find that direct contact with L. donovani amastigotes induces the formation of primarily actin-based long tubular membrane protrusions by the B cells. While the formation of short membrane protrusions and spreading of the B cells are associated with B cell activation (17), we observed the protrusions formed upon exposure to the parasite to be longer and able to connect two cells. Similar structures, called tunneling nanotubules (TNTs), have been identified in many different cell types, including immune cells, such as T cells and macrophages, and were shown to be involved in intercellular trafficking of various cargos, ranging from nucleic acids and proteins to whole organelles (reviewed in reference 20). B cells were also found to be capable of forming tunneling nanotubules among themselves (27), with T cells (39), and with macrophages (40). These B cell TNTs were shown to enable the transport of plasma membrane proteins on the outside of the tubules as well as to allow passage of microvesicles through the inside of the tubules (39, 41). In other cell types, previous studies have demonstrated these tubules to be exploited for disease propagation by bacteria, including Listeria monocytogenes (21), Mycobacterium bovis (22), and Chlamydia trachomatis (23), and viruses, such as HIV-1 (24), influenza A virus (25), and SARS-CoV-2 (26). Thus, the involvement of these TNT-like protrusions in the dissemination of L. donovani among B cells represents the first report of this mechanism of intercellular communication in the context of a parasitic infection.

Interestingly, we find L. donovani present in the splenic B cell area as early as 14 days postinfection, evidencing the direct contact between B cells and the parasite *in vivo*, with a visible increase in the number of parasites found at 28 days, which coincides with the loss of germinal centers in the spleen during the chronic stages of visceral leishmaniasis (42). This is in line with previous findings of B cells from the spleens of infected mice carrying parasite *ex vivo* (16). How these parasites come to be in the B cell area is not yet understood. One possible mechanism is the transfer of parasites from macrophages, which represent the main target for L. donovani and populate the marginal zone of the spleen along with the MZB, to B cells. Indeed, we find that macrophages and B cells are capable of transferring L. donovani bidirectionally and that this transfer can be facilitated by the formation of TNT-like connections between the two immune cell types. This is supported by a previous study in which TNTs have been shown to be formed between macrophages and B cells, where they aided in the transport of the immunosuppressive protein negative factor (Nef) involved in the pathogenesis of HIV and the development of immune dysfunction (40). As MZB have been shown to be the predominant B cells to carry L. donovani *in vivo* (16) and we consistently see MZB capturing the parasite to a higher degree *in vitro* in this work, marginal zone shuttling represents a plausible mechanism through which parasites could be transported into the B cell area of the spleen after parasite transfer from macrophages to MZB. A similar shuttling mechanism in which ligation of CD21 causes MZB to migrate into the white pulp has been described to deliver antigen (43) or IgM-containing immune complexes (44) to follicular B cells. Indeed, we have found CD21 to play a key role in capturing the parasite and inducing TNTs. This is further supported by the fact that amastigotes passaged in *Rag1*^−/−^ mice are coated in C3 (16), whose fragments are ligands for CD21, the complement receptor 2 (29). An additional consideration as to how the parasite could be exchanged between macrophages and B cells is its localization within the cells. While *Leishmania* resides in parasitophorous vacuoles within the macrophages, B cells do not fully internalize the parasite but rather carry it in actin-based cup-like structures on their surface (16). Thus, a transfer of parasites between these cell types might require the parasite to exit the macrophage. A possible mechanism previously identified for L. amazonensis which may be at play here is the transfer of amastigotes enclosed in host macrophage membranes extruding from the cells (45). These LAMP1/LAMP2-rich structures can thus facilitate the exchange of parasites at a close range without full lysis of the macrophage. How the parasite can induce B cell activation after capture, however, remains to be fully elucidated. We have previously reported that hypergammaglobulinemia in L. donovani infection is exacerbated by an IFN-I-dependent upregulation of endosomal TLRs (10) and that, although the role of type I IFNs seems to depend on the strain of *Leishmania* and the experimental procedure used, IFN-Is play a negative role in mouse models of L. donovani infection (46). While positive feedback loops of IFN-I signaling leading up to upregulation of TLR7 and -9 in B cells have been previously proposed (47, 48), the presence of an early IFN-I wave that could signal B cells to induce a detrimental upregulation of endosomal TLRs remained to be demonstrated. In this work, we show that the presence of the parasite induces a very early, transient expression of both *Ifna* and *Ifnb* mRNA, which we show to induce IFN-I signaling in the B cells within 1 h of exposure to the parasite. Hence, this early production of IFN-I could induce the upregulation of endosomal TLRs, which in turn aggravate the disease by increased cytokine and antibody production; however, the pathway of induction of this early IFN-I remains to be elucidated, as B cells have been shown capable of IFN-I production induced via different pathways, such as (endosomal) TLRs and cytosolic sensors (49). Furthermore, the extent to which other signaling pathways are synergistic with, or parallel to, the enhanced endosomal TLR signaling leading up to polyclonal B cell activation remains to be elucidated in future studies.

In summary, we demonstrate the dissemination of L. donovani between B cells via the use of CD21-induced TNT-like protrusions, thus propagating B cell activation. Additionally, the transfer of amastigotes can occur bidirectionally and partially via TNT-like protrusions. This may lead to parasite acquisition by MZB from macrophages in the marginal zone and subsequent shuttling to the B cell area, ultimately leading to polyclonal B cell activation and hypergammaglobulinemia.

## MATERIALS AND METHODS

### Mice and parasites.

C57BL/6 and B6.SJL-Ptprc^a^ Pepc^b^/BoyJ (referred to as CD45.1) congenic mice were purchased from The Jackson Laboratory and housed under specific-pathogen-free conditions at the Laboratoire National de Biologie Expérimentale (LNBE) following guidelines for good animal practice provided by the Canadian Council on Animal Care. *Ifnar1*^−/−^ mice were a kind gift from Alain Lamarre (INRS-CAFSB). Animals were used at 8 to 12 weeks of age, following protocols approved by the Animal Care and Use Committee of the Centre Armand-Frappier Santé Biotechnologie (protocol numbers 1910-01 and 2002-03).

Leishmania donovani (strain LV9) was maintained by serial passage in B6.129S7-Rag1^tm1Mom^ (*Rag1^−^*^/−^) mice, and amastigotes were isolated from the spleens of infected animals. Mice were infected by intravenous injection of 2 × 10^7^ amastigotes via the lateral tail vein.

For *in vitro* experiments using previously frozen amastigotes, parasites were opsonized using fresh serum from *Rag1*^−/−^ mice at 37°C for 30 min prior to use. Leishmania donovani promastigotes (strain LV9) were grown by axenic culture of amastigotes isolated from the spleens of *Rag1*^−/−^ mice at 26°C in M199 medium (Sigma) supplemented with 10% heat-inactivated fetal bovine serum (FBS; Wisent), hypoxanthine (Sigma), hemin (Sigma), biopterin (Sigma), biotin (Sigma), and penicillin-streptomycin (Life Technologies). The resulting promastigotes were washed in Dulbecco’s phosphate-buffered saline (DPBS), opsonized or not with serum from *Rag1*^−/−^ mice as described above, and finally resuspended in complete Iscove’s modified Dulbecco’s medium (IMDM) prior to use. Toxoplasma gondii tachyzoites (strain RH) were maintained in Vero cells by serial passage in Dulbecco’s modified Eagle’s medium (DMEM) (Gibco, Invitrogen) supplemented with 5% heat-inactivated FBS (Wisent) and supplemented with penicillin-streptomycin (Life Technologies) and HEPES (Fisher) as previously published (50). Prior to experiments, the tachyzoites from Vero cells were harvested by scraping the cells, any clumps were disrupted by passage through a 27G needle, and cell debris was pelleted and removed by centrifugation at low speed (200 × *g*). The egressed tachyzoites in the supernatant were pelleted by high-speed centrifugation (1,300 × *g*), resuspended in ice-cold phosphate-buffered saline (PBS) (pH 7.2 to 7.4), and filtered from debris using a 3-μm polycarbonate filter (Millipore). The resulting tachyzoites were then washed using PBS and finally resuspended in complete DMEM prior to the experiment.

### Fluorescent labeling of parasites.

After isolation or rapid thawing, the amastigotes to be labeled were washed in plain RPMI medium (Gibco, Invitrogen) and in DPBS (Mg^2+^ and Ca^2+^ free; Gibco, Invitrogen) before resuspension in 100 μL diluent C (Sigma) per 10^8^ amastigotes. An equivalent volume of diluent C containing 1:250 PKH67 membrane linker dye (Sigma) was added to the suspension and incubated for 3 min at room temperature. The staining reaction was stopped by adding an equivalent volume to the total amount of diluent C of FBS to the parasites and incubating at room temperature for 1 min. The thus-stained parasites were washed in plain RPMI medium and complete IMDM before use in subsequent experiments.

### *In vitro* B cell culture.

Naive splenic B cells were purified using a negative selection magnetically assisted cell sorting kit (Miltenyi Biotech) according to the manufacturer’s protocol and cultured in IMDM (Gibco, Invitrogen) containing 10% heat-inactivated FBS (Wisent) and supplemented with penicillin-streptomycin (Life Technologies). Purified B cells were then incubated by themselves, with the parasite at a multiplicity of infection (MOI) of 5 or 10, or with 10 ng/mL mouse recombinant IFN-β (BioLegend) at 37°C and 5% CO_2_ for the duration specified. For experiments assessing the necessity of direct contact for B cell activation, transwell permeable membrane inserts (Corning) with a pore size of 0.4 μm and a polyethylene terephthalate (PET) membrane were used to separate total B cell populations or B cell subpopulations, and only the cells in the top compartment were exposed to PKH67-labeled parasite.

### Differentiation of bone marrow-derived macrophages (BMM).

To obtain macrophages, bone marrow was flushed from the femurs and tibias of C57BL/6 mice, and red blood cells were lysed using a solution containing 0.17 M NH_4_Cl, pH 7.4, for 7 min and cultured in adherent culture-treated petri dishes in DMEM (Gibco, Invitrogen) supplemented with 10% heat-inactivated FBS, 10 mM HEPES, pH 7.4, and penicillin-streptomycin (Life Technologies) supplemented with 20% L929 cell-conditioned medium (LCM) at 37°C. After 1 day, nonadherent cells were collected and transferred to new petri dishes, and differentiation was restimulated by the addition of LCM on days 3 and 5 after bone marrow collection. On day 7, cells were detached from the dish surfaces using a sterile cell scraper, counted, and reseeded on sterile coverslips at a concentration of 2.5 × 10^5^ macrophages per slip in DMEM containing 10% FBS for confocal microscopy experiments or in 12- or 24-well-plates at concentrations of 1 × 10^6^ or 5 × 10^5^, respectively, for flow cytometry experiments and allowed to adhere for 16 h overnight before use.

### Parasite transfer experiments.

To assess parasite transfer between parasite-exposed cells and naive cells, B cells or macrophages were isolated or differentiated from both wild-type C57BL/6 mice expressing CD45.2 and from CD45.1 congenic mice as described above. The cells were seeded in 24-well plates at a concentration of 1 × 10^6^, and the wells containing cells from one allelic variant were exposed to PKH67-labeled LV9 amastigotes for 1 h for B cells or overnight in the case of macrophages, thoroughly washed using sterile PBS for a minimum of three times until there was no presence of free parasite visible under the microscope, and then further incubated with naive cells of the other allelic variant for 2.5 or 5 h before preparation of samples for flow cytometry or confocal microscopy. For experiments using cytochalasin D, the parasite-exposed cells were treated with 1 or 10 μM cytochalasin D (Sigma) for 1 h before further coincubation with the initially nonexposed cells of the other allelic variant for 2.5 or 5 h. The absence of free parasites in the supernatant was further investigated by subjecting naive cells to the supernatants of exposed cells after washing.

### Cross-linking of surface markers using antibody-coupled beads.

Latex beads with a mean particle size of 3 μm (Sigma) were washed with sterile DPBS (Gibco), resuspended in DPBS, and incubated with 1 μg per 10^7^ beads of CD21/CD35 monoclonal antibody (clone 4E3; eBioscience), IgM rat anti-mouse antibody (clone II/41; BD Biosciences), or a combination of both for 2 h at room temperature under continuous mixing on a rotating wheel. As controls, beads were either uncoupled or coupled to the isotype control antibody rat IgG2a kappa isotype control (clone eBR2a; eBioscience). After incubation, the antibody-coupled beads were washed and resuspended in complete IMDM (Gibco, Invitrogen) before incubation with the B cells at an MOI of 7 for 5 h.

### Quantitative real-time PCR (RT-qPCR).

RNA extraction from isolated B cells was carried out using the RNeasy minikit (Qiagen) following the manufacturer’s instructions. Reverse transcription was performed using the iScript cDNA synthesis kit (Bio-Rad) per the manufacturer’s protocol. Real-time PCR measurements were prepared using iTaq Universal SYBR green supermix (Bio-Rad) and carried out using a Stratagene mx3005p real-time PCR system. Genes for IFN-α, IFN-β, and hypoxanthine phosphoribosyltransferase (HPRT) were amplified using primers listed in Table 1. Data were normalized to HPRT and expressed as the fold increase relative to naive controls.

**TABLE 1:** Primer sequences for RT-qPCR

Gene	Direction	Sequence
*Hprt*	Fw	5′-GTT GGA TAC AGG CCA GAC TTT GTT G-3′
	Rv	5′-GAT TCA ACC TTG CGC TCA TCT TAG GC-3′
*Ifna*	Fw	5′-CAT CTG CTG CTT GGG ATG GAT-3′
	Rv	5′-TTC CTG GGT CAG AGG AGG TTC-3′
*Ifnb*	Fw	5′-TCA GAA TGA GTG GTG GTT GC-3′
	Rv	5′-GCA CTT TCA AAT GCA GTA GAT TCA-3′

The primers in Table 1 were used to measure the relative gene expression using RT-qPCR.

### Confocal microscopy.

L. donovani amastigotes were stained with the PKH67 membrane linker dye kit (Sigma) following the protocol described above prior to incubation with splenic B cells or macrophages. For experiments using only B cells, the cells were seeded at 2 × 10^6^ cells per well in 12-well plates and incubated either alone or with PKH67-stained L. donovani amastigotes (at an MOI of 5:1 or 10:1) for 2.5, 5, or 8 h before fixation in a final concentration of 2% paraformaldehyde for 10 min at room temperature. To prepare cells for intracellular staining, if applicable, permeabilization was achieved by treating fixed cells using 0.1% Triton X-100 in PBS and unspecific binding of antibodies was blocked using PBS containing 5% bovine serum albumin (BSA), 5% FBS, and a 1:200 dilution of FcBlock antibody (clone 2.4G2) for 30 min at room temperature. The cells were then labeled with a combination of the following antibodies as specified: anti-IgM-AF568 (polyclonal; Invitrogen), phalloidin-AF594 (Invitrogen), antitubulin-AF647 (clone 10D8; BioLegend), anti-CD45.1-BV421 (clone A20; BioLegend), anti-CD45.2-AF647 (clone 104; BioLegend), anti-CD11b-BV421 (clone M1/70; BD Biosciences). NucBlue (Hoechst 33342; Invitrogen) was used to stain cell nuclei following the manufacturer’s protocol. Labeled cells were washed in PBS and resuspended in PBS and then transferred onto coverslips coated with poly-l-lysine (Sigma-Aldrich), before being mounted on microscopy slides using Fluoromount-G mounting medium (Invitrogen).

In experiments using macrophages and B cells, sterile coverslips were treated with poly-l-lysine, and macrophages adhered directly onto the coverslips before being incubated either directly with PKH67-stained LV9 amastigotes, followed by naive B cells, or with B cells previously incubated with PKH67-stained parasites. Coverslips were incubated for 5 h after the addition of B cells to ensure adherence of B cells to the coverslips before fixation, blocking, staining, and mounting as described above.

### Immunohistochemistry.

Spleens of naive and infected C57BL/6 mice were collected on days 21 and 28 postinfection and snap-frozen in blocks of clear OCT compound (Fisher Health Care) using liquid nitrogen. Blocks were stored at −80°C until being sectioned into 10-μm slices using a Microm HM525 cryostat (Thermo Fisher) at −0°C. Tissue sections were mounted on glass slides and immediately processed by air drying and rehydrating tissues in PBS for 30 min. Unspecific staining was blocked by treatment of tissues with PBS containing 5% BSA and 1:100 of FcBlock antibody (2.4G2) for 1 h. LV9 amastigotes were stained by incubating tissues with serum from LV9-infected hamsters at a concentration of 1:500 and then labeling bound anti-LV9 using an anti-hamster-AF488 conjugate (BioLegend). Tissue cells were labeled using CD11b-BV421 (BD Biosciences) and IgM-AF568 (Invitrogen) and subsequently fixed using 1% paraformaldehyde (PFA) before mounting coverslips on Fluoromount-G mounting medium (Invitrogen) over the tissue. Images were taken on an LSM780 confocal microscope (Zeiss) using either a 40× or 63× oil immersion objective.

### Western blotting.

Total cell protein extracts from 1 × 10^7^ purified B cells were lysed in radioimmunoprecipitation assay (RIPA) buffer (Sigma-Aldrich) supplemented with a protease inhibitor cocktail (Complete mini; Roche) and a phosphatase inhibitor cocktail (phosSTOP; Roche). Protein concentration of lysates was determined following the instructions of a Pierce bicinchoninic acid (BCA) protein assay kit (Thermo Fisher), and equal amounts of protein (15 μg) were separated on an 8% SDS-PAGE gel before semidry transfer to a nitrocellulose membrane (Hybond; Amersham). Proteins were labeled using a Phospho-Stat1 (Tyr701, clone 58D6) rabbit monoclonal antibody or a Stat1 (D1K9Y) rabbit monoclonal antibody (both Cell from Signaling Technology). Blots were cut between 76 and 52 kDa as determined by the travel of the Amersham ECL Rainbow Full-Range Molecular Weight marker (stripping buffer; Thermo Fisher), and equal loading was confirmed with a monoclonal antibody against β-actin (Santa Cruz Biotechnology).

### Flow cytometry.

The presence of PKH67-stained LV9 on various B cell subsets was assessed using the following antibodies: anti-CD21-phycoerythrin (PE)-CF594 (clone 7G6; BD Biosciences), anti-CD23-allophycocyanine (APC) (clone B3B4; BioLegend), anti-CD45.1-PE (clone A20; BD Biosciences), anti-CD45.2-APC-Cy7 (clone 104; BioLegend), anti-CD86-BV510 (clone GL1; BD Biosciences), and anti-MHCII-BV421 (clone M5/114.15.2; BD Biosciences). To conserve the phosphorylation status during the measurement of type I IFN signaling, cells were fixed in 2% PFA prior to permeabilization using cold methanol and staining using the following antibody panel: anti-CD21-PE-CF594 (clone 7G6; BD Biosciences), anti-CD23-PE-Cy7 (clone B3B4; BD Biosciences), anti-MHCII-fluorescein isothiocyanate (FITC) (clone 2G9; BD Biosciences), and anti-pSTAT1-AF647 (clone 4a; BD Biosciences). For each sample, 100,000 cells were acquired using an LSRFortessa cell analyzer (BD), and data were analyzed using FlowJo software (BD).

### Statistical analysis.

Statistical analysis was done in GraphPad Prism using Student’s *t* test. Differences were considered statistically significant at a *P* value of <0.05. All experiments were independently carried out at least three times.

### Data availability.

All data generated during this study are included in this article.
