# Supplementary material for: Leishmania donovani Exploits Tunneling Nanotubes for Dissemination and Propagation of B Cell Activation
Source: Microbiol Spectr. 2023 Jul 5;11(4):e05096-22. doi: 10.1128/spectrum.05096-22 (PMC10434010; doi:10.1128/spectrum.05096-22)
Supplement: Supplemental file 1 — Supplemental material. Download spectrum.05096-22-s0001.pdf, PDF file, 2.9 MB [file spectrum.05096-22-s0001.pdf]

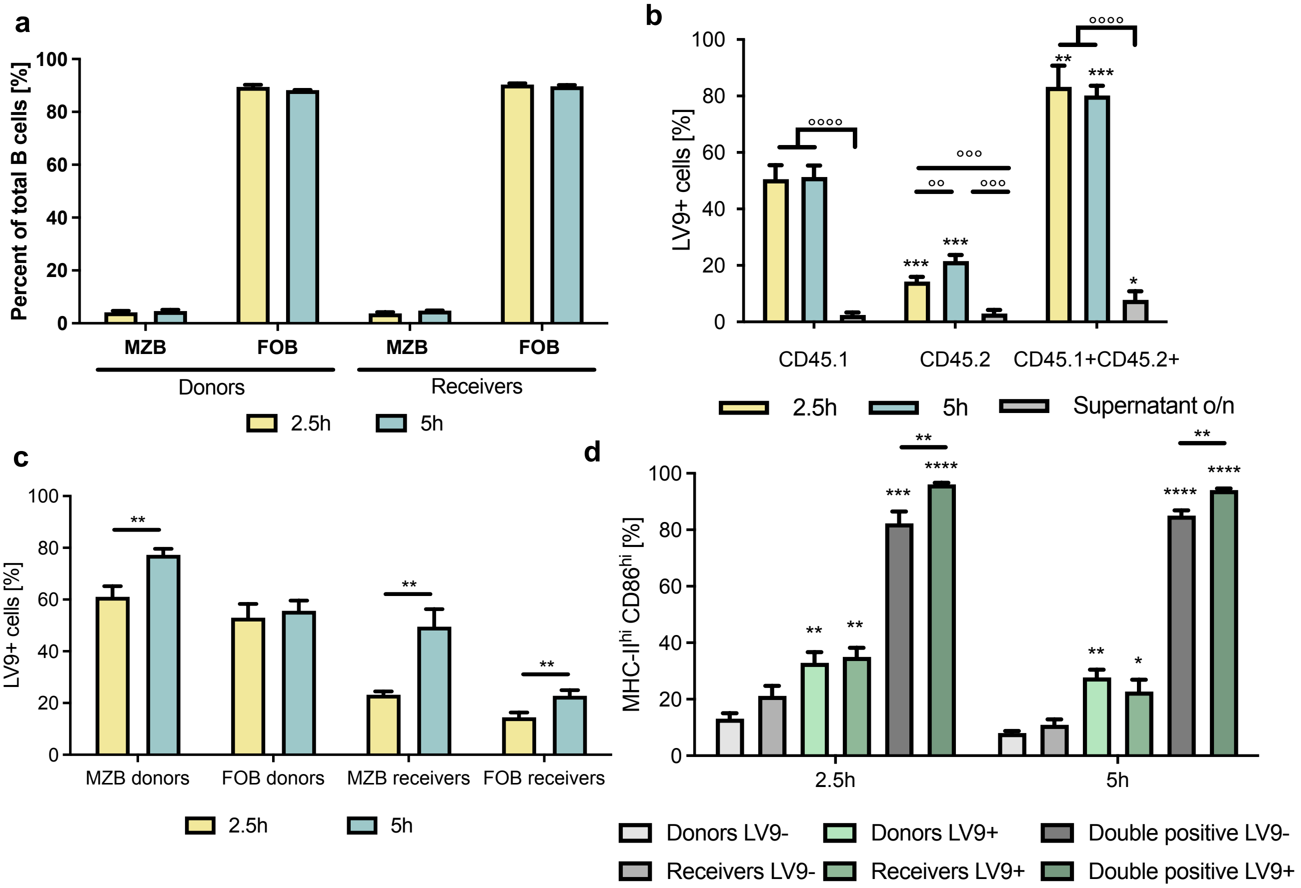

Supplemental Figure 1. (a) B cells purified from CD45.2 mice were exposed to *L. donovani* for 1h before thorough washing to remove uncaptured parasites and exposure to naïve B cells purified from CD45.1 for 2.5h or 5h. Cells were separated into donors (CD45.1^{+}) and receivers (CD45.2^{+}) and within those groups, the percentages of cells falling into the MZB (CD21^{hi} CD23^{lo}) and FoB (CD21^{lo} CD23^{hi}) subgroups were assessed by flow cytometry. (**b-d**) Inversely, B cells purified from CD45.1 mice were exposed to *L. donovani* for 1h before thorough washing to remove uncaptured parasites and exposure to naïve B cells purified from CD45.2 for 2.5h or 5h. (**b**) Percentage of cells carrying PKH67-stained positive amastigotes in cells gated on their expression of CD45.1 or CD45.2 as measured by flow cytometry. (**c**) Percentage of MZB (CD21^{hi} CD23^{lo}) and FoB (CD21^{lo} CD23^{hi}) cells carrying parasite within the CD45.1^{+} donor and CD45.2^{+} receiver groups. (e) Percentage of cells expressing high levels of CD86 and MHCII within the CD45.1^{+} donors, CD45.2^{+} receivers, and CD45.1^{+}CD45.2^{+} clusters carrying parasite (LV9^{+}) or not (LV9^{-}). For all experiments, B cells were exposed to parasites with an MOI of 1:10. Data represented as mean ± SD from one of three independent experiments. *$p < 0.05$, **$p < 0.01$, ***$p < 0.001$, ****$p < 0.0001$.

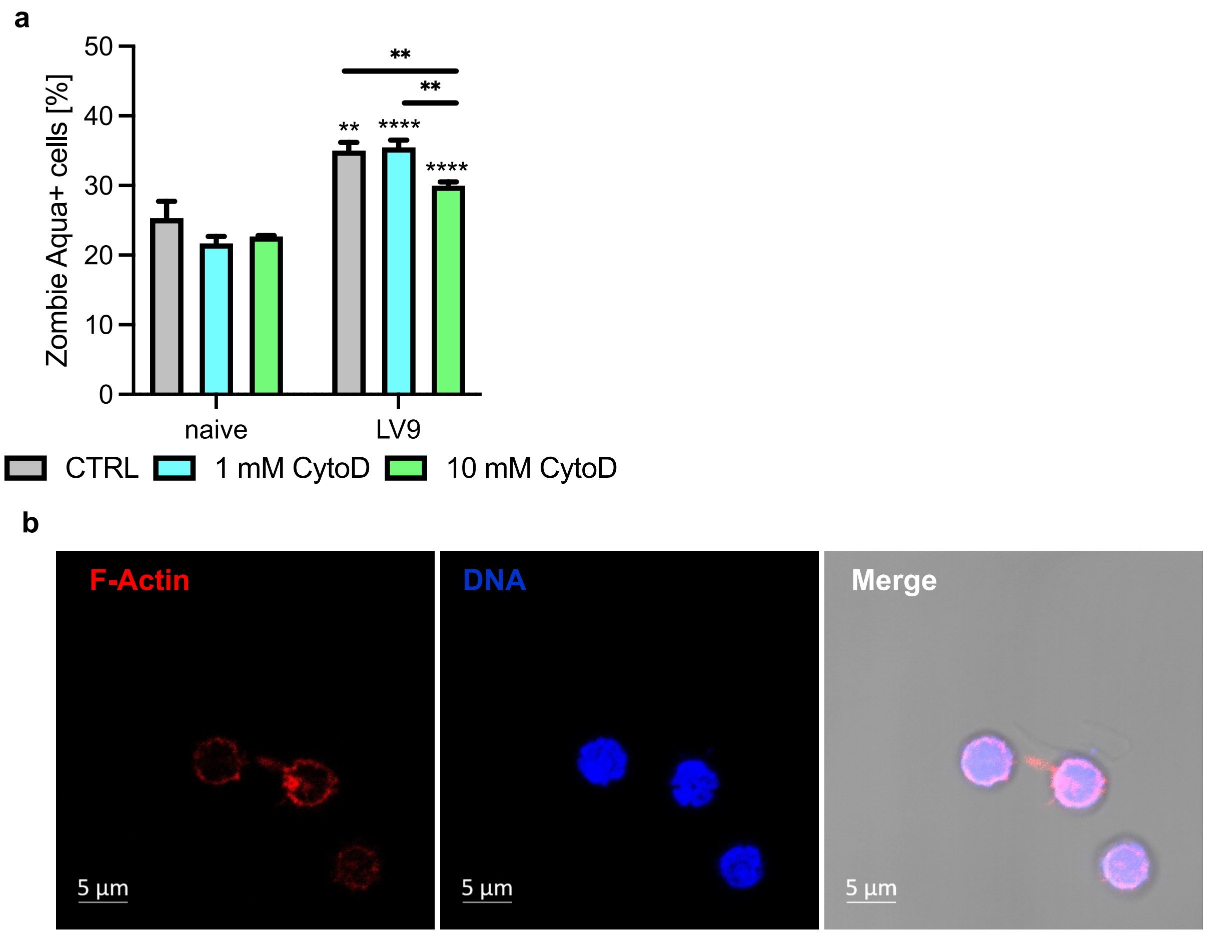

Supplemental Figure 2. (**a**) B cells purified from CD45.1 mice were exposed to *L. donovani* (MOI 1:10) for 1h before thorough washing to remove uncaptured parasites and treatment with 1 or 10 µM Cytochalasin D, prior to co-incubation with B cells from CD45.2 origin for 2.5 or 5 h. (**b**) B cells were exposed to opsonized promastigotes at an MOI of 7 for 5h and labeled using phalloidin-AF594 (F-actin, red) and Hoechst33342 (blue).

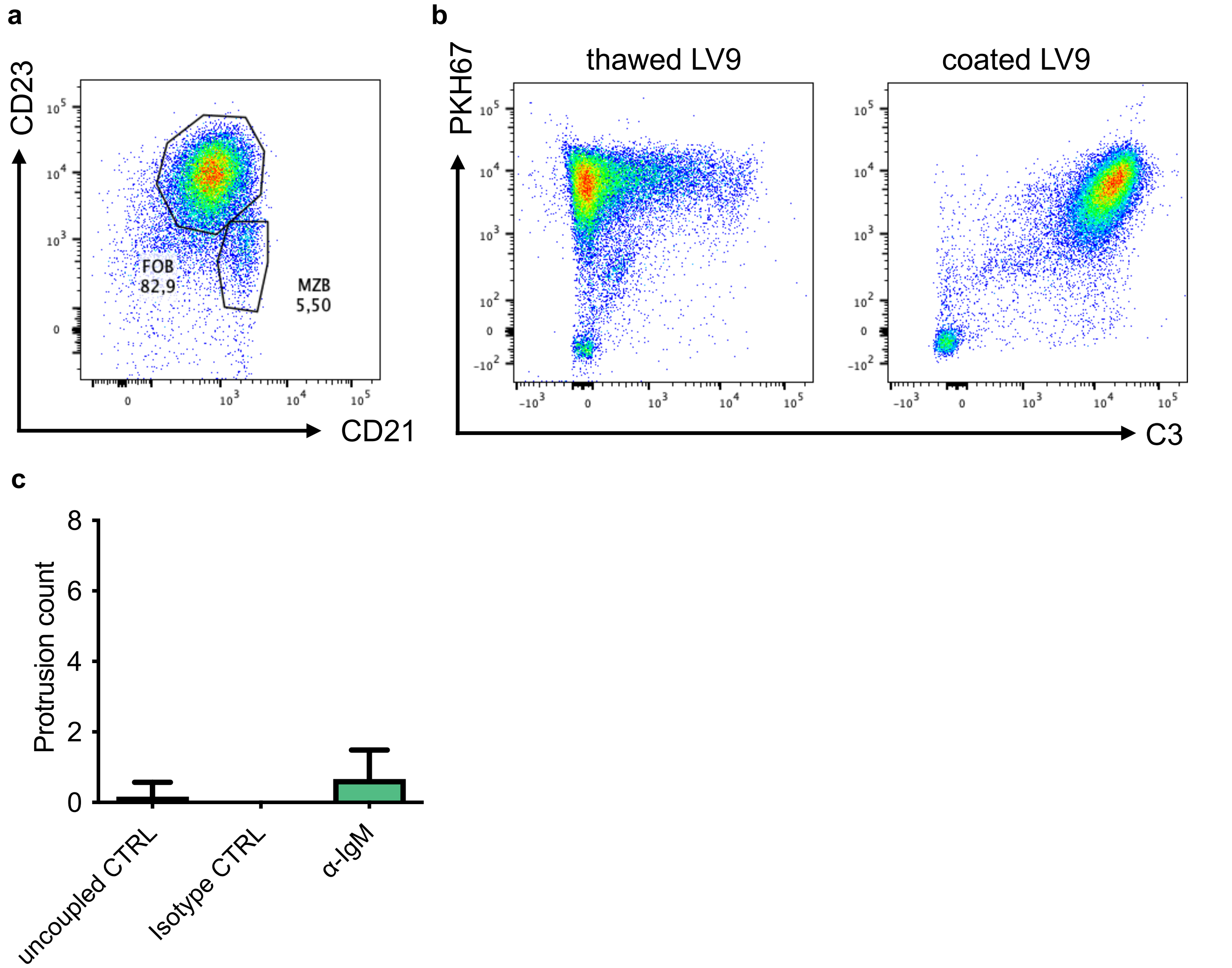

Supplemental Figure 3. (**a**) Representative FACS plots showing CD21 and CD23 expression on purified splenic B cells. (**b**) Representative FACS plots displaying complement C3 and PKH67 staining of *L. donovani* amastigotes that were previously frozen in liquid nitrogen. Plot on the left represents parasites that were simply thawed and PKH67-stained (frozen LV9), plot on the right shows parasites that were additionally treated with serum from *Rag1*$^{-/-}$ mice. (**c**) Antibodies against IgM or a Rat IgG2a isotype control were coupled to latex beads and exposed to B cells for 5h. Protrusions between B cells with or without parasites were counted over 70 microscope fields per sample. Data represented as mean ± SD *$p < 0.05$, **$p < 0.01$, ***$p < 0.001$, ****$p < 0.0001$.

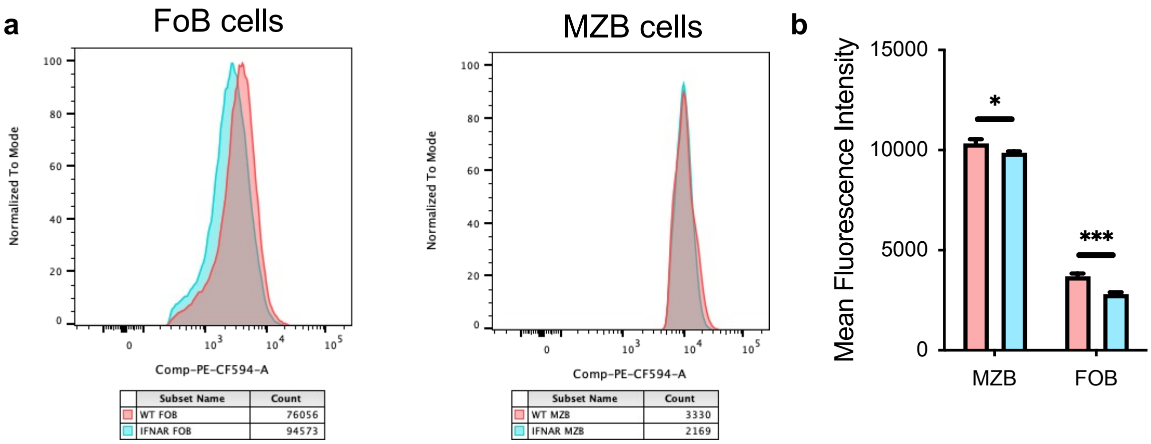

Supplemental Figure 4. (**a**) Representative FACS histograms showing CD21 expression in FoB (left) and MZB (right) cells. The cells from the WT mouse are shown in red, those from the *Ifnar*$^{-/-}$ mouse are in blue. (**b**) Mean fluorescence intensity of CD21 in MZB and FOB from WT (red) and *Ifnar*$^{-/-}$ (blue). Data represented as mean ± SD. *$p < 0.05$, **$p < 0.01$, ***$p < 0.001$.

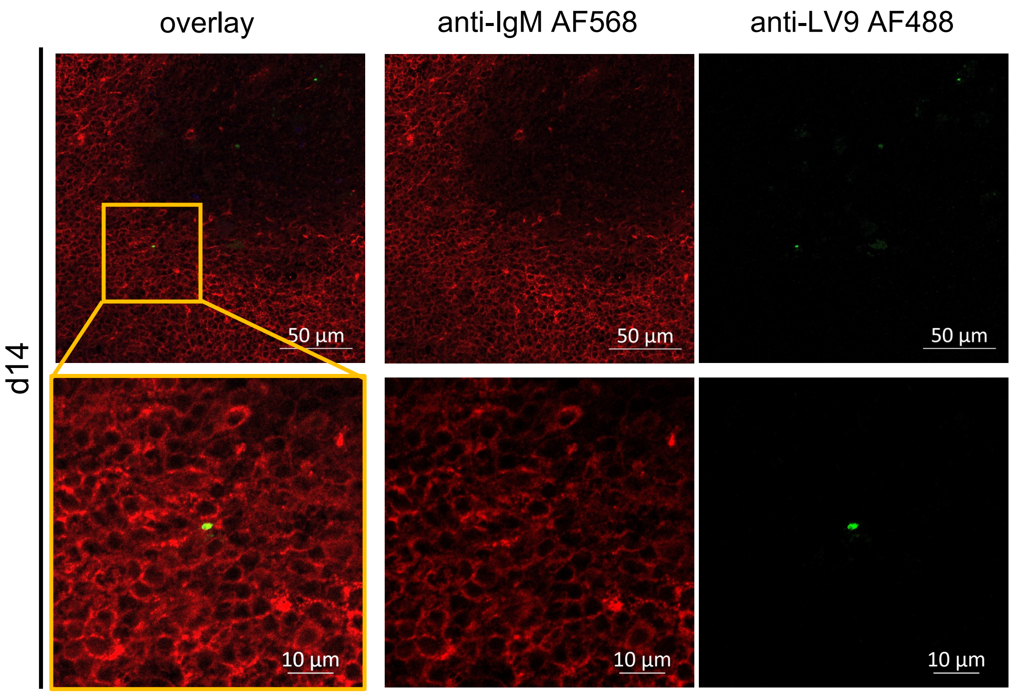

Supplemental Figure 5. *L. donovani* can be seen in the splenic B cell area of infected mice as early as day 14 post-infection. Splenic cryosections of *L. donovani*- infected mice at day 14. The first row was taken using a 40x oil immersion objective while the second row represents an image taken using a 63x objective at the location specified using a yellow square. Tissue sections were stained using IgM-AF568 (red) and the parasite was stained by incubation with serum from *L. donovani*-infected hamsters and secondary anti-Hamster-AF488 antibody (green).

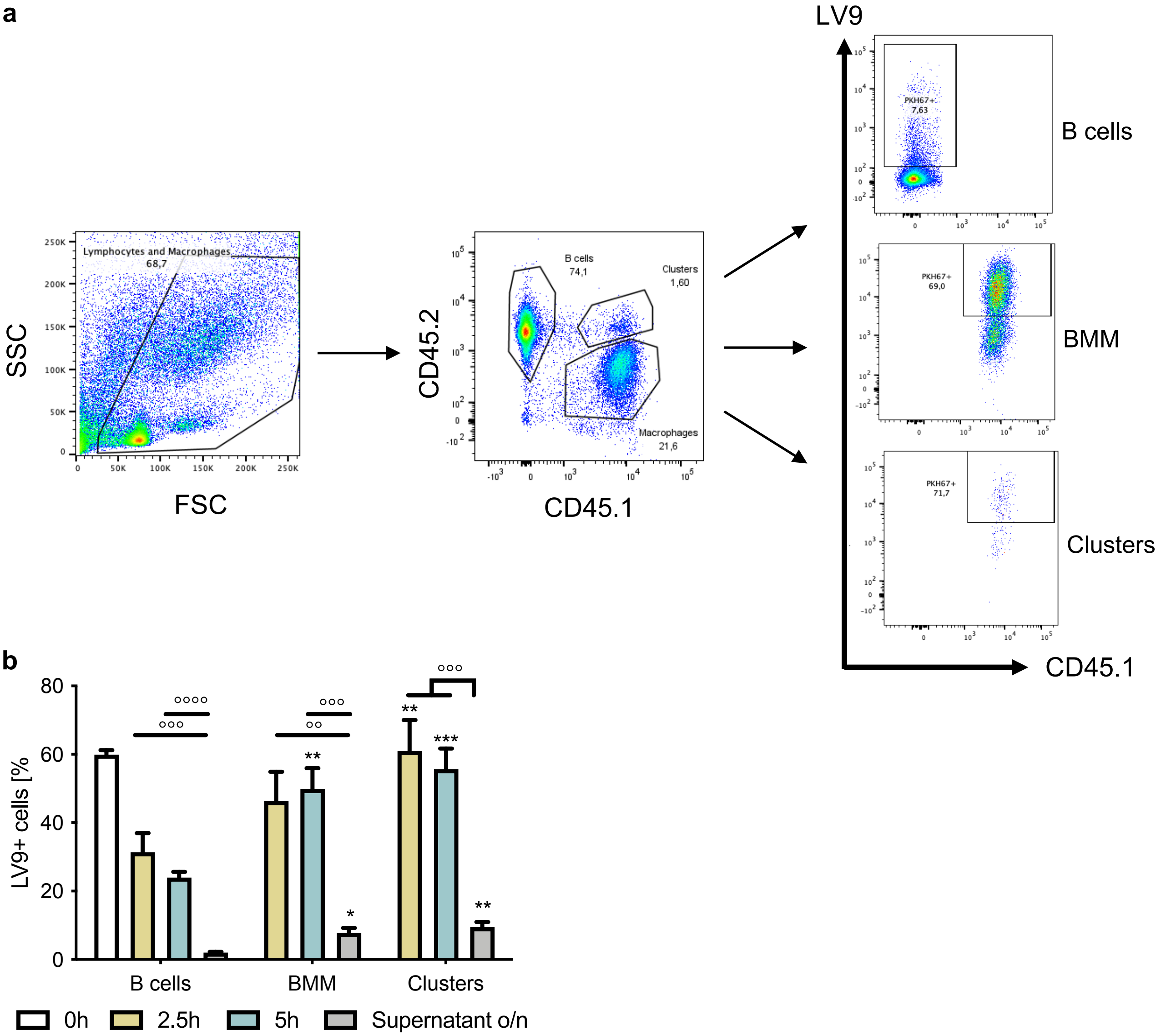

Supplemental Figure 6. (**a**) Macrophages derived from the bone marrow of CD45.1 mice (BMM) were exposed to *L. donovani* (MOI 1:10) overnight before thorough washing to remove uncaptured parasite and exposure to naïve B cells purified from CD45.2 for 5h at a 1:1 ratio. Representative FACS plots showing gating strategy and percentages of B cells, macrophages, and clusters carrying parasite. (**b**) B cells purified from CD45.2 mice were exposed to *L. donovani* (MOI 1:10) for 1h before thorough washing to remove uncaptured parasite and exposure to CD45.1$^+$ BMM at a 1:1 ratio. Percentage of cells carrying PKH67-stained amastigotes in cells differentiated into CD45.2$^+$ B cells, CD45.1$^+$ BMM, or CD45.1$^+$CD45.2$^+$ clusters as measured by flow cytometry. Data represented as mean ± SD from one of four independent experiments. *$p < 0.05$, **$p < 0.01$, ***$p < 0.001$, ****$p < 0.0001$.
